# The current status and future development of high-temperature conventional superconductivity

**DOI:** 10.1093/nsr/nwae047

**Published:** 2024-02-24

**Authors:** Mikhail I Eremets

**Affiliations:** High Pressure Chemistry and Physics Group, Max-Planck-Institut für Chemie, Germany

## Abstract

The robust evidence and reproducibility of high-temperature superconductivity in hydrogen-rich materials under challenging experimental conditions of megabar pressures is presented.

This survey highlights key advancements in high-temperature superconductivity in hydrogen-rich materials, emphasizing the robust evidence and reproducibility of superconductivity under challenging experimental conditions of megabar pressures. In the near future, achieving room-temperature superconductivity is highly probable, and the field is expected to transition towards near-ambient-pressure superconductivity.

A new family of superconductors, hydrogen-rich superconductors, was established following the discovery of superconductivity (SC) with a critical temperature (*T_c_*) of 203 K in hydrogen sulphide H_3_S compressed to megabar pressures [1]. H_3_S is a covalent metal with strong bonds between sulphur and hydrogen atoms. Many others are hydride-rich hydrides or superhydrides with a very different structure, in which hydrogen atoms are weakly covalently bonded to form a cage, while the host metal atom is in the centre of the cage and acts as an electron donor to the hydrogen network. A record *T_c_* of ∼250 K has been achieved in LaH_10_ [2]. These hydrides are conventional superconductors, allowing the calculation of superconducting properties in conjunction with crystal structure predictions. The original ideas must be credited to Neil Ashcroft, who first (1968) realized that high-*T_c_* conventional SC was theoretically possible in metallic hydrogen [2], and 40 years later (2004) also guessed that metallic hydrogen sublattices could be stabilized at a more accessible pressure in H-rich materials [2].

The new superconductors such as H_3_S [1–5], YH_9_ [6–8] and LaH_10_ [2] are stable at high pressures, ∼140–200 GPa. Even higher pressures of ∼350–400 GPa are required for the expected room-temperature SC in MgH_6_, LaH_18_, CeH_18_ and several other materials. Despite the limitations imposed by the high-pressure environment, successful synthesis, structural characterization and experimental evidence of SC at these pressures have been achieved.

Superhydrides are produced at megabar pressures by reacting pure metals with excess hydrogen, often using laser heating. The use of alternative hydrogen sources, such as ammonia borane and hydrocarbons, has greatly simplified the experiments.

The primary tool for the detection and characterization of SC is electrical measurements. Four probe electrical transport measurements provide *T_c_* from a sharp change in the temperature dependence of the electrical resistance *R(T)* (Fig. 1a) and the true zero resistance in the superconducting state. The dependence of *T_c_* on the applied magnetic field gives the value of the upper critical field *H_c2_* at which SC is destroyed, and hence the coherence length ξ (Fig. 1b).

**Figure 1.**
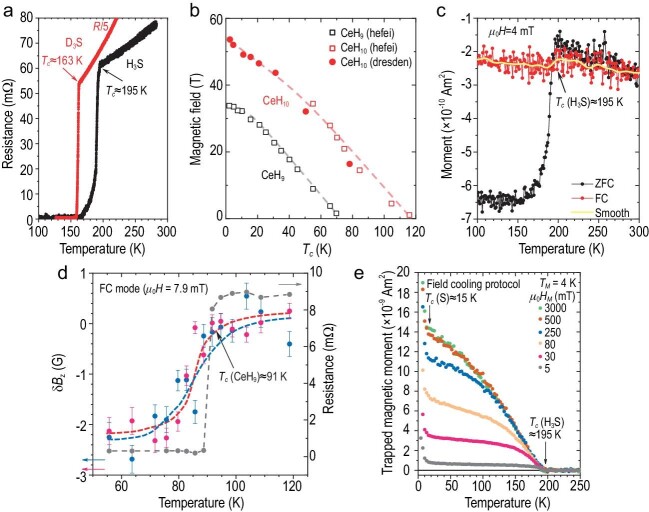
Characterization of superconducting state in hydrogen-rich compounds using different methods. (a) Four-probe electrical transport measurements demonstrate a sharp resistance drop to zero Ohm in both H_3_S (at 141 GPa) and its deuterated counterpart D_3_S (at 154 GPa) (details are in ref. [17]). (b) The dependence of *T_c_* on applied magnetic field in CeH_9_ and CeH_10_. In both cerium hydrides, SC is suppressed at magnetic fields exceeding *H_c2_* (data from ref. [14]). (c) Magnetic susceptibility measurements in H_3_S at 162–167 GPa (ref. [18]). The Meissner effect in superconducting H_3_S at 155 GPa (magnetic field expulsion upon cooling the sample below its *T_c_* at an applied magnetic field, field cooling (FC) mode—red circles) is noticeably weaker than the magnetic field screening in zero field cooling (ZFC) mode (black circles). The yellow curve represents smoothed FC data (Fast Fourier Transformation (FFT) filter, 7 points) for clarity. (d) The Meissner effect is rather pronounced in CeH_9_ as found using the NV centre technique (red and blue dots), consistent with simultaneous measurements of electrical resistance indicating a transition at *T_c_* ≈ 91 K at 137 GPa (grey dots) data from ref. [10]). (e) Temperature dependence of a trapped magnetic moment in superconducting H_3_S generated under FC conditions at different magnetic fields *μ_0_H_M_* (5–3000 mT) (details are in ref. [9]).

Magnetic susceptibility measurements are equally important (see for example, reference [9]) as they provide a wealth of information about a superconductor, including *T_c_*, lower critical field *H_c1_*, penetration depth *λ_L_*, vortex creep, critical current density and other parameters (Fig. 1c). These measurements are even more challenging than electrical measurements but are currently possible up to at least 200 GPa using a tiny non-magnetic diamond anvil cell (DAC) coupled with a SQUID magnetometer [1]. The coil technique is also employed [3], and a new magnetometry method based on nitrogen vacancy (NV) centres has recently been used [10]. The latter revealed a pronounced Meissner effect in CeH_9_ [10] (Fig. 1d).

However, the Meissner effect is subtle or barely observable in H_3_S and LaH_10_ (Fig. 1c). This is not surprising for superconductors with strong vortex pinning. In the presence of strong pinning, the interesting phenomenon of trapped magnetic flux can be observed [9]: after a magnetic field is removed, a remnant magnetization persists up to *T_c_* (Fig. 1e). This phenomenon provides conclusive evidence for SC as such. The trapped flux method is highly effective when combined with a DAC, as the background from the DAC is virtually eliminated since the signal is measured in nominally zero applied magnetic fields. Further evidence for SC in H_3_S is provided by the shielding of applied magnetic fields by the H_3_S sample, monitored by nuclear magnetic resonance scattering of synchrotron radiation with a ^119^Sn Mössbauer sensor. Finally, the superconducting gap in H_3_S was estimated by infrared spectroscopy [2] and tunnelling spectroscopy (to be published).

The isotope effect, i.e. the shift in *T_c_* resulting from the replacement of hydrogen by deuterium atoms, provides evidence for the electron-phonon mechanism of SC in hydrides. A significant shift in *T_c_* was observed in H_3_S, LaH_10_, YH_6_, YH_9_, CeH_9_ and CeH_10_ [11], as well as in other compounds (Fig 1a).

It is important to note that SC in hydrogen-rich compounds, such as H_3_S [1,3–5], LaH_10_ [2], YH_9_ and YH_6_ [6–8], CaH_6_ [12,13], CeH_9_ and CeH_10_ [10,14], has been successfully reproduced and confirmed by several independent research groups. Importantly, the crystal structure of these compounds has been thoroughly characterized, ensuring reliable comparisons of results between different research teams.

Powerful theoretical predictions of crystal structures and calculations of SC have greatly accelerated the experimental search for new superconductors [2]. There are numerous examples of excellent agreement with experimental observations, such as the prediction of SC in LaH_10_ at 280 K at ∼200 GPa [2], which closely matches the experimentally observed *T_c_* of ∼250 K at 170 GPa [2]. Another recent example is CaH_6_ at *T_c_* ∼ 215 K, where the remarkable cage structure was predicted long before it was realized in other hydrides ([13] and references therein). These and other successes may create the illusion that experiments merely confirm predictions, and that the main challenge is to select suitable materials from a large number of candidates. However, the importance of experimental research should not be underestimated. First, it is the ultimate judge of the validity of new superconductors. Secondly, even when driven by theoretical predictions, experiments often reveal unexpected phenomena.

For example, in the case of H_2_S, SC at 70 K was predicted and confirmed, but it was serendipitously discovered that H_2_S can be transformed into H_3_S, which has *T_c_* = 203 K. Independently, SC with *T_c_* = 200 K was predicted in H_3_S at high pressure in another system (H_2_S)_2_H_2_. Experiments often reveal stable and metastable phases that have been overlooked in theoretical calculations (as observed in LaH_10_). Deviations from the predictions also highlight the importance of anharmonic and other corrections. In a number of cases, the predicted phases have not been found yet (H_3_Se, YH_10_, MgH_6_, LuH_6_, HfH_10_, etc).

All these discrepancies do not necessarily mean that the predictions are wrong; rather, the calculations can be refined based on these experimental results. However, publishing negative results is a challenge. It is clear that the synergy between theory and experiment needs to be strengthened through direct contacts and discussions, especially as the field moves from binary to ternary systems in the search for new superconductors, not only at high, but also at low and ambient pressures. The numerous predictions of superconductors with *T_c_* > 80 K ([15,16] and references therein) hold great promise for practical applications.

Finally, the tantalizing goal of achieving room-temperature SC appears to be within reach, given the predictions of numerous phases in the currently accessible pressure range of 300–400 GPa.
